# Validation data for the determination of perchlorate in water using ion chromatography with suppressed conductivity detection

**DOI:** 10.1186/s12302-016-0086-5

**Published:** 2016-06-07

**Authors:** Maike A. Seiler, Detlef Jensen, Udo Neist, Ursula K. Deister, Franz Schmitz

**Affiliations:** 1 Hessian State Laboratory, Glarusstr. 6, 65185 Wiesbaden, Germany; 2 Thermo Fisher Scientific, Im Steingrund 4-6, 63303 Dreieich, Germany; 3 Hochschule RheinMain, Am Brückweg 26, 65428 Rüsselsheim, Germany

**Keywords:** Perchlorate, Contamination, Disinfection by-products, Ion chromatography, Method validation

## Abstract

**Background:**

Perchlorate salts are relatively stable, soluble in water, and migrate into groundwater sources. Groundwater is an essential source for drinking water suppliers. Perchlorate bears health risks as it is identified to impair normal thyroid function by interfering with iodine uptake by the thyroid gland. The development of a sensitive analytical method for the determination of perchlorate is therefore of the highest interest or public health. Ion chromatography is a sensitive method suitable for perchlorate determinations. This manuscript describes the validation of an ion chromatographic method. Perchlorate is determined by ion chromatography (IC) with conductivity detection after suppression (CD) applying isocratic elution.

**Results:**

In this study, the suitability of IC-CD was tested for synthetic samples, selected environmental water, drinking water, and swimming pool water in order to evaluate potential matrix effects on the perchlorate signal even after sample preparation. A sample injection volume of 750 μL was applied to the selected 2-mm-IC column. In untreated samples, the perchlorate peak can be interfered by neighbouring signals from matrix ions like chloride, nitrate, carbonate, and sulphate. Depending on the concentration of the matrix ions, the perchlorate peak can show asymmetric shape in particular when the perchlorate concentration is low. Recovery is reduced with increasing matrix ion concentrations. Dedicated matrix elimination was applied to minimize such effects. A reporting limit of 1.5 μg/L perchlorate and an expanded measurement uncertainty of 13.2 % were achieved.

**Conclusion:**

The extended method validation proves the applicability of IC based on the EPA 314.0 method for the determination of trace amounts of perchlorate in water samples of different origin. The results support the development of a respective international standard pursued by ISO. The approach evidenced its working robustness and ease of use in terms of eluent preparation, chromatographic resolution, column life time and sample preparation. Due to the simplified analytical workflow of the analytical procedure the application’s integration into the collection of methods of interested laboratories should be facilitated.

## Background

Perchlorate is identified as an environmental contaminant and found in drinking, ground, and surface waters, with its origins being both anthropogenic and natural. In its natural origin, perchlorate was found to originate from atmospheric deposition, possibly from chloride aerosol being exposed to an electrical discharge in the presence of ozone [1]. In Chile saltpetre [2], perchlorate is found as a natural impurity, and it is also of anthropogenic origin due to its use as solid propellant for rockets, missiles, fireworks, pyrotechnics, flares, matches, ordnance, and explosives [3]. Perchlorate is of toxicological interest because it impairs normal thyroid function by interfering with iodine uptake by the thyroid gland and the production of important thyroid hormones [4, 5]. Iodine-containing hormones are essential for early childhood development [6]. Hypothyroidism, a disorder of the endocrine system in which the thyroid gland does not produce enough thyroid hormone, in early stages of pregnancy bears the risk for impaired physical and mental development of the human foetus [7].

In 2011, the US Environmental Protection Agency (EPA) determined that perchlorate meets the federal Safe Drinking Water Act’s (SDWA) three criteria for regulating a contaminant: (1) perchlorate may adversely affect public health; (2) there is a substantial likelihood that perchlorate frequently occurs in public water systems at levels of health concern, as monitoring data show that over 4 % of public water systems have detected perchlorate, and (3) there is a meaningful opportunity for health risk reduction for the 5.2–16.6 million people who may be getting drinking water that contains perchlorate [8].

The Interim Lifetime Drinking Water Health Advisory of the EPA recommends a provisional advisory drinking water limit of 15 µg/L perchlorate [9], with California having an enforceable standard with a maximum contaminant level of 6 μg/L perchlorate [10]. Analytical results from 3870 US drinking water samples in 2005 revealed perchlorate concentrations between 4 and 420 µg/L in 4.1 % of the samples [11]. European interest in the determination of perchlorate was triggered by a pollution of the Bordeaux-drinking water with perchlorate [12] and recent findings of it in food and beverages in the mg/kg-range [13].

In Germany, perchlorate was detected in groundwater close to intensively farmed areas in North Rhine-Westphalia with a maximum value of 5.8 µg/L. In swimming pool water samples perchlorate levels reached the values as high as 980 µg/L [14] and in pore water samples from the “Maifeld” area in Berlin with the values up to 15,000 µg/L sampled immediately after firework displays [15]. The German Federal Environment Agency (UBA) is in discussions regarding the addition of perchlorate to the parameter list in the technical regulations for the testing standard of inline electrolysis plants used for drinking water and swimming pool water disinfection [16].

Perchlorate contamination of agricultural soils and products using already polluted surface and groundwater sources for irrigation may be assumed. In addition, after-crop disinfection techniques for fruit and vegetable treatment where perchlorate can be formed as a by-product could be an additional source for perchlorate in food [11]. Early in 2015, the European Commission released a statement regarding the presence of perchlorate in food in which the EU Member States, with the active involvement of food business operators, were requested to monitor the presence of perchlorate in food [17].

The determination of perchlorate is therefore of the highest interest for public health. As a result several standardization organizations have developed methods for the determination of perchlorate in matrices addressing the uptake through food and most importantly through drinking water.

Several analytical approaches are already published to determine trace perchlorate in aqueous samples. Perchlorate can be analysed using IC-CD, applying heart-cutting techniques, or IC-ESI/MS. All of these techniques are already standardized by the EPA. EPA 314.0 [18], EPA 314.1 [19], and EPA 314.2 [20], all use IC-CD. EPA 314.0 describes a direct injection method with or without previous off-line matrix elimination sample treatment for perchlorate determinations, while EPA 314.1 and EPA 314.2 require a combined inline concentration and matrix elimination step using heart-cutting techniques. Specific for EPA 314.1 is the necessity to apply a secondary confirmation column with different chromatographic selectivity to validate the perchlorate results obtained with the first separator column. The different chromatographic characteristics of the concentrator and separator columns to be used in accordance EPA 314.2 prevent the use of the secondary confirmation step. The methods of the EPA 314 series allow perchlorate determinations in the low µg/L range. EPA 331.0 applies IC-ESI/MS and can be applied for the determination of perchlorate in the low ng/L range [21]. Currently, an ISO working group is developing an internationally applicable standard for water quality control based on suppressed conductivity detection with and without heart-cutting techniques [16].

In our work, we focussed on perchlorate determinations that are being carried out by direct sample injection without heart-cutting techniques using standard analytical IC equipment, addressing the laboratory workflow needs, as well as the analytical and financial requirements of future users. This should promote a broader acceptance of the planned standard method, thus facilitating its integration in the method collection of interested laboratories. This article refers to extended validation experiments based on the EPA 314.0 method in support of the ISO development work.

## Experimental

### Reagents

The water used fulfilled the requirements of ISO 3696, Grade 1 [22] and had a specific resistance of 18.2 MΩ•cm (Milli-Q Reference A+, Merck Millipore, Darmstadt, Germany). The anion stock standard solutions (1 g/L) for chloride, sulphate (Merck KGaA, Darmstadt, Germany), and perchlorate (CPAchem, Stara Zagora, Bulgaria and SCP Science, Courtaboeuf, France) were of p.a. quality. The perchlorate solution from CPAchem was used for the method validation and calibration experiments, while the perchlorate solution from SCP Science served as an independent source for quality control measurements.

### Materials for sample preparation

Disposable 10-mL-syringe Inject Solo on polypropen/polyethene basis (Braun Melsungen AG, Melsungen, Germany), disposable non-sterile cellulose acetate syringe filter (0.45 µm, 25 mm, VWR international, Darmstadt, Germany), and three layer OnGuard II Ba/Ag/H polymer-based cation exchanger cartridges (Thermo Fisher Scientific, Dreieich, Germany) were used. In addition to the producer’s instructions [23], each new cartridge needed to be rinsed with 15 mL of water in the upside down position before use in order to remove any gas bubble. The ion exchange cartridges were operated in vertical direction using a Supelco 12-port-Visiprep SPE vacuum manifold before sample feed. Each of the samples was applied to the ion exchange cartridge at a maximum flow rate of 2 mL/min to ensure an optimal ion exchange [23]. The first 6 mL fraction of the filtered sample was discarded. The following fraction was used for the analysis. Tests for blank values, adsorption, and memory effects were investigated for comparison with the filter materials. All of the cartridges checked showed no blank signal, perchlorate losses, or memory effects for water or synthetic samples.

### Instrumentation

The chromatographic instrument (Thermo Scientific Dionex DX-500) consisted of a gradient pump (Dionex GP50) with a flow of 0.25 mL/min, an autosampler (Dionex AS50) with 1000-µL-injection loop, a column thermostat set at 30 °C (Dionex Ultimate 3000 TCC-3000), an eluent generator (Dionex RFC-30) equipped with an eluent generator cartridge (Dionex EGC-III KOH), continuously regenerated trap column (Dionex CR-ATC), and a conductivity detector (Dionex CD-25). The entire flow path was metal free. For eluent suppression prior to conductivity detection, a Dionex AERS 500 was used at a current setting of 22 mA. Separation was performed on a Dionex IonPac AS20 column and guard column-set. Columns and suppressor were in the 2-mm format, and data acquisition and evaluation were done using the Dionex Chromeleon 6.70 chromatography software. The eluent (35 mmol/L KOH) was produced electrolytically in situ. Samples were injected with volumes between 500 and 1000 µL using partial loop injection for volumes below 1000 µL. The total runtime was 30 min for each sample.

### Analysis

Statistical performance data, calibration approach, and the working conditions were evaluated based on ISO standard methods [24, 25] for a concentration decade (e.g. 1 to 10 µg/L) analysing 10 standard solutions of different perchlorate concentrations. For routine analysis, a minimum of 5 concentration levels were calibrated each day of operation for the defined working range. For both, a first-order calibration was applied. All other samples and standards were injected in replicate (n = 3). Although a number of values were identified as outliers according to Grubbs-test, no value was eliminated for the following calculations. Sample dilution can be applied as long as the perchlorate concentration of the injected sample does not fall below the lowest calibration standard defining the working range. Both peak area and peak height integration were used.

## Results and discussion

### Chromatographic conditions

The eluent and column were chosen to provide both fast elution and best chromatographic resolution for perchlorate while at the same time ensuring the lowest possible noise in suppressed conductivity detection facilitating a trace perchlorate determination at low µg/L-concentrations.

### Analytical working range

For the given concentration decade, each of the 10 calibration solutions was injected using different injection volumes. Table 1 summarizes the statistical results obtained for peak evaluation using peak area and height integration.

**Table 1:** Comparison of performance data obtained applying various injection volumes

Performance data 1 µg/L to 10 µg/L	Injection volume and signal evaluated					
	500 µL		750 µL		1000 µL	
	Area	Height	Area	Height	Area	Height
Linearity of the function	Yes	Yes	Yes	Yes	Yes	Yes
*s*_x0_ (µg/L)	0.128	0.106	0.105	0.171	0.0603	0.0659
*V*_x0_ (%)	2.33	1.93	1.90	3.12	1.10	1.20
LOD (µg/L)	0.512	0.424	0.420	0.684	0.241	0.264
LOQ (µg/L)	1.54	1.27	1.26	2.05	0.724	0.791

*s*_*x0*_ standard deviation of the procedure [24]; *V*_*x0*_ Variation coefficient of the procedure (relative *s*_x0_) [24]; *LOD* approximate value for the limit of detection (LOD = 4·*s*_x0_, [25]); *LOQ* approximate value for the limit of quantification (LOD = 12·*s*_x0_, [25])

Depending on the samples’ ionic strength, defined by the concentrations of the major ions like chloride, nitrate, carbonate, and sulphate, perchlorate elutes on a drifting baseline and can be integrated as a ‘rider peak’ by the chromatography software. The evaluation of ‘rider peaks’ can prove to be difficult and can possibly lead to erroneous results [26]. EPA 314.0 specifies that sample pre-treatment can be effective as a means to eliminate certain matrix interferences [18].

Initial experiments, examining matrix effects, involved replicate injections of a perchlorate-free local drinking water that was spiked with 1 and 5 µg/L perchlorate using injection volumes between 500 and 1000 µL. The reported peak areas and peak heights were compared to those obtained injecting diluted standard solutions of the same perchlorate concentrations using identical conditions. Table 2 summarizes the respective data.

**Table 2:** Perchlorate peak area and peak height recovery in drinking water using different injection volumes

Injection volume (µL)	Drinking water^a^ + 1 µg/L ClO_4_^−^		Drinking water^a^ + 5 µg/L ClO_4_^−^	
	Rec^b^ peak area (%)	Rec^b^ peak height (%)	Rec^b^ peak area (%)	Rec^b^ peak height (%)
500	85.4	74.5	91.7	85.9
600	73.8	75.3	88.3	87.8
700	67.9	70.9	85.1	84.8
800	67.7	69.8	83.4	81.2
900	60.7	65.5	82.2	78.7
1000	55.7	50.3	79.5	64.0

^a^Source: Wiesbaden, Germany. Matrix: chloride: 36 mg/L; sulphate: 68 mg/L; nitrate: 2.8 mg/L—determined by IC

^b^Rec: recovery of the perchlorate signal in the drinking water sample

Rec Peak Area = 100 × Area(Sample)/Area(Standard); Rec Peak Height = 100 × Height(Sample)/Height(Standard)

**Fig. 1:**
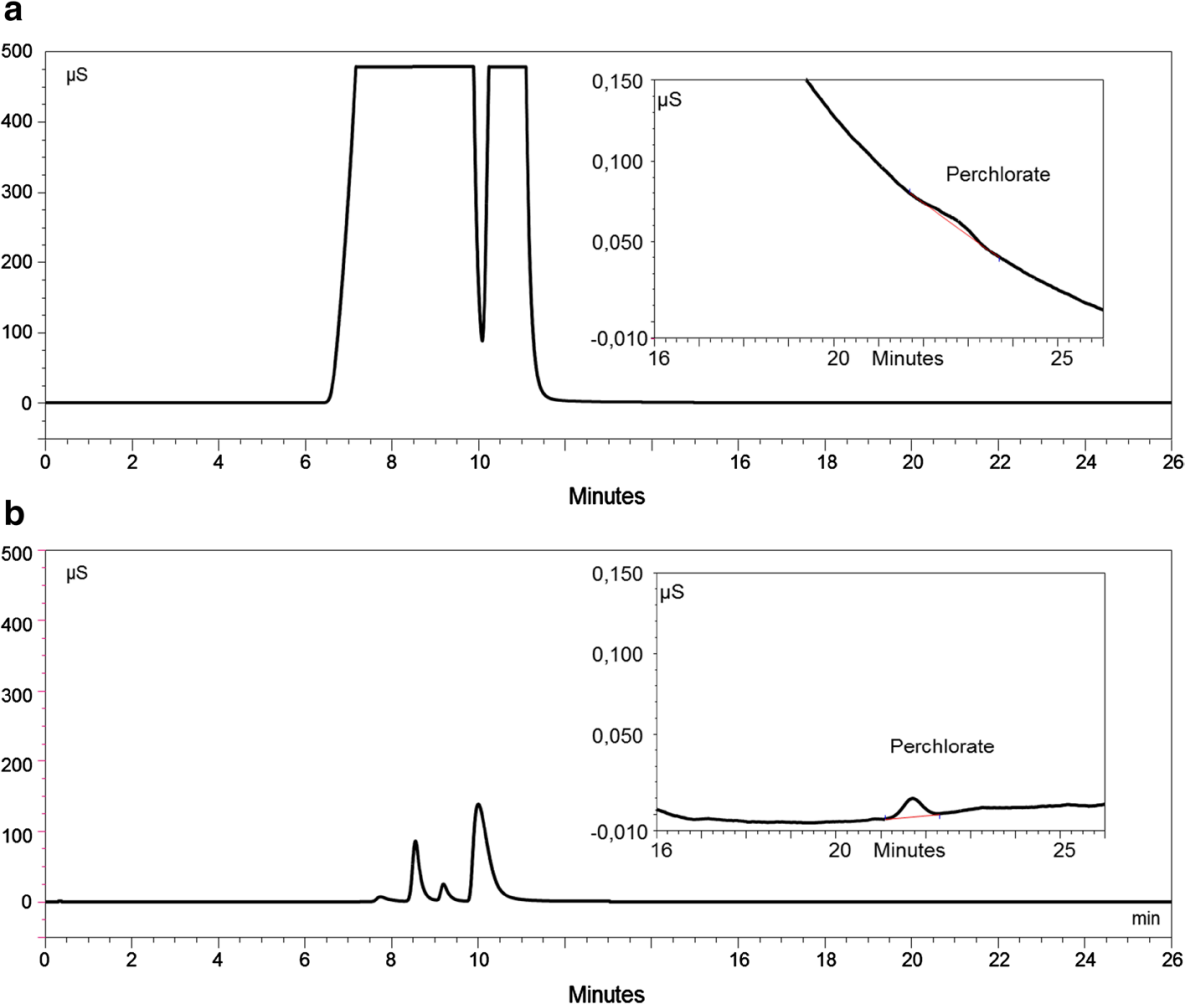
Influence of sample preparation—comparison of treated and untreated samples spiked with 3 µg/L perchlorate. Trace (**a**) untreated surface water (660 mg/L chloride, 260 mg/L sulphate, 14 mg/L nitrate); Trace (**b**) treated surface water

For both concentration levels, a negative trend for peak area recovery and peak height recovery was obtained along with increasing injection volumes. The perchlorate peak becomes a rider peak on the tailing flank of the sum peak obtained for the matrix anions—a representative chromatogram can be seen in Fig. 1. For both concentration levels, a similar decline is obtained. The overall impact is different depending on the analyte concentration. It appears that recovery at lower concentrations is lower compared to the respective experiment at higher concentration. The data that are displayed in Tables 1 and 2 helped to the decision of using a working range from 1.5 to 15 µg/L perchlorate and an injection volume of 750 µL. Using these conditions, *V*_x0_ was determined at 1.0 %; LOD was 0.33 µg/L and LOQ resulted in 0.98 µg/L.

### Sample preparation

Chloride, sulphate, and carbonate, as well as metals can be removed using cation exchangers in the Ag-, Ba-, and H-form [23]. The Ba-form resin needs to be activated either by calcium ions present in the sample or with a calcium chloride solution added to the sample to ensure that barium ions are available for the precipitation reaction with sulphate. As long as the chromatographic resolution between perchlorate and sulphate does not fall below R = 1.3 [27], no need arises to add calcium chloride solution. Regardless of this possibility, the addition of calcium chloride solution was carried out in this study for all samples with unknown calcium loads.

Slingsby et al. [28] investigated the use of a resin cartridge train in the order OnGuard-Ba, OnGuard-Ag, and OnGuard-H and evaluated the recovery of several oxyanions on a 10 mg/L concentration level (Slingsby RW, personal communication) for the tested oxyanions. As perchlorate was not on their list similar experiments were performed using the three layer OnGuard-Ba/Ag/H cartridge [23]. Perchlorate solutions at different concentration levels (1, 1.5, and 5 µg/L perchlorate) in water without matrix ions and in synthetic solutions containing a combination of 100 or 500 mg/L of chloride and sulphate each were treated and analysed. The perchlorate signal obtained from an untreated perchlorate standard solution served as reference for the calculation of the recovery rates (Table 3).

**Table 3:** Recovery of perchlorate in synthetic matrix solutions after preparation

Sample	Matrix-free standard; direct injection	Matrix-free standard^a^	100 mg/L chloride/sulphate^a^	500 mg/L chloride/sulphate^a^
1.0 µg/L ClO_4_^−^				
Rec^b^ area (%)	100 ± 1.19	88.8 ± 0.28	83.8 ± 4.6	84.7 ± 4.4
Rec^b^ height (%)	100 ± 0.89	91.3 ± 0.41	91.3 ± 3.5	88.9 ± 2.9
1.5 µg/L ClO_4_^−^				
Rec^b^ area (%)	100 ± 0.32	102 ± 4.9	86.0 ± 4.1	106 ± 4.5
Rec^b^ height (%)	100 ± 0.49	101 ± 3.7	87.6 ± 1.9	110 ± 4.0
5.0 µg/L ClO_4_^−^				
Rec^b^ area (%)	100 ± 0.76	96.1 ± 1.9	95.7 ± 2.6	97.0 ± 2.0
Rec^b^ height (%)	100 ± 0.76	98.8 ± 0.84	96.6 ± 0.82	98.7 ± 0.71

^a^pre-treated with three layer cartridge

^b^Rec: recovery of the perchlorate signal in sample [Rec Peak Area = 100 × Area(Sample)/Area(Standard); Rec Peak Height = 100 × Height(Sample)/Height(Standard)]

Recoveries obtained were in the range of 84 to 110 % for the chloride and sulphate spiked samples. These data were within the range EPA 314.0 [18] defined for the recovery acceptance criterion of 85 to 115 %. In contrast to the producer’s recommendation, the cartridges were applied to more than one sample. The criterion applied was the chromatographic resolution (*R*) between perchlorate and the next eluting peak (*R* ≥ 1.3). As long as this criterion was fulfilled, the chromatogram was integrated and the cartridge was used. In case of lower resolution—an indirect indication of lower matrix ion removal due to the exhaustive use of the cartridge—the original sample was treated with a new cartridge. The effect of sample preparation can be seen in Fig. 1, comparing the chromatogram of an untreated and the one of a treated sample. The removal of matrix anions proved to be beneficial for both the chromatographic behaviour as well as the recovery (Table 3).

### Equivalency of approaches

In order to estimate the possible influence of the cartridge treatment on the perchlorate result, the equivalency of the analytical data obtained by direct sample injection (fundamental analytical procedure) and of the data obtained after injecting the sample after treatment (overall analytical procedure) the method of orthogonal regression described in [29] was used. Possible constant-systematic deviations were tested by the joined *t-*test and for potential proportional-systematic deviations the *Chi-square*-test was applied. Both statistical evaluations were applied to the perchlorate calibration (1.5 to 15 µg/L) for peak areas and peak heights. No proportional-systematic or constant-systematic deviations were found, proving the statistical equivalency of non-treated sample measurement and treated sample measurement. Due to this result, a calibration of the fundamental analytical procedure could be applied to all of the measurements for this study.

### Stability of perchlorate control and standard solutions

Although perchlorate solutions are widely considered stable, reports indicate the possibility of reductive decomposition under anaerobic conditions [30, 31]. Consequently, the stability of aqueous perchlorate solutions (1.5 and 7.5 µg/L) stored in white glass flasks at ambient temperature on the laboratory bench (temporarily exposed to sunlight) was monitored by IC over 8 weeks. Deviation from the expected value, as defined in [32, 33], was below the accepted limit of ±10 % for 14 days for the 1.5 µg/L and for 57 days for the 7.5 µg/L standard solution. Following these procedures, no significant changes of the 7.5 µg/L standard solutions were obtained during the entire period investigated.

### Expanded measurement uncertainty for the working range 1.5 to 15 µg/L

The measurement uncertainty was estimated according to ISO 11352 [34] due to a requirement of ISO/IEC 17025 [35]. Precision data for the calculation of random uncertainty components were taken from control charts, from replicate analyses, and calibration experiments. Systematic uncertainty components were calculated from the mean recovery of the 7.5 µg/L control standard made from the 1000 µg/L SPC Science reference solution considering the indicated uncertainty of the material.

Random and systematic uncertainty contributions were merged for the calculation of the combined measurement uncertainty *u*_c_. Multiplication of *u*_c_ by 2 gave a resulting expanded measurement uncertainty of 13.2 %.

### Retention time stability

The possible influence of large sample volume injections on the performance of the separator system has been tested with previously unused Dionex IonPac AG/AS 20 columns. The perchlorate retention times of a 7.5 µg/L standard solution were recorded over 34 days, yielding more than 1000 injections. During this time, the perchlorate retention time decreased by 2.5 min indicating a loss of anion exchange capacity of the separator column. As large sample volumes (up to 1000 µL) of real-world samples—including waste and surface waters, some of which were untreated—had to be injected in order to achieve the desired LOD and LOQ, it is to be expected that larger amounts of other matrix components, like non-polar organics, humic acids, or transition metals, were co-injected, leading to the decrease in retention time. It is therefore advisable to check the performance of the guard column regularly and replacing it at an early stage, if necessary [36] to extend the separator column’s lifetime.

Modern ion chromatography standard methods [37] allow a deviation of retention time up to ±10 % within a batch. During the studies of this work perchlorate retention times met this requirement even for a much longer period and remained in these limits for more than 30 days of continuous operation with more than 1000 injections. The decrease in retention time did not show significant influence on the performance data *V*_x0_, which was evaluated on each workday following the procedure described in [33]. All *V*_x0_ values calculated were below 3.33 % fulfilling the requirements described in [33].

### Analytical results

The method was applied to the determination of perchlorate in arbitrarily selected real-world samples including drinking, raw, swimming pool, surface, and waste waters of different origins. Samples with perchlorate concentrations below 1.5 µg/L were spiked resulting in added concentrations of 1.5 and 3 µg/L of perchlorate, respectively. The samples were prepared as described above. Only the swimming pool water samples and one of the surface water samples had to be diluted into the calibrated perchlorate working range before analysis. The method was not applicable for all of the six waste water samples (clarified sewage), though they were additionally treated with non-polar RP C18 cartridges. The chromatograms showed noisy and non-reproducible drifting baselines or signals in the retention time window of perchlorate, which could not reliably be identified as the target peak even after standard addition experiments. Remarkably, the interfering components were exclusively observed in waste water samples of domestic and industrial origin. They are anionic—as they are detected after suppressed conductivity—strongly retained on the stationary phase used and not removed by the combination of cation exchange and non-polar SPE. There is a high likelihood that those unknowns might be organic acids of complex composition.

Table 4 summarizes the results and the corresponding contents of matrix ions.

**Table 4:** Perchlorate contents in real samples

Sample	Matrix	Perchlorate (µg/L)	Matrix ions		
			Chloride (mg/L)	Nitrate (mg/L)	Sulphate (mg/L)
1	Raw water	<LOD	11	0.09	48
2	Raw water	1.6	130	27	64
3	Raw water, spiked	0.93 ± 0.44	24	200	46
4	Raw water	1.7	59	150	240
5	Raw water	<LOD	140	<0.01	46
6	Raw water, spiked	0.58 ± 0.28	56	41	77
7	Drinking water	<LOD	45	3.0	59
8	Drinking water	<LOD	32	3.1	77
9	Drinking water	3.4	60	24	140
10	Drinking water	<LOD	22	6.4	17
11	Drinking water	<LOD	33	2.8	74
12	Drinking water, spiked	<1.5	81	44	20
13	Surface water, DF 75	900	n. d.	n. d.	n. d.
14	Surface water	<LOD	660	14	260
15	Surface water, spiked	<1.5	60	12	29
16	Surface water, spiked	0.29 ± 0.16	35	16	54
17	Surface water, spiked	0.39 ± 0.38	39	16	51
18	Surface water	<LOD	23	12	19
19	Surface water	9.3	86	18	130
20	Surface water, spiked	< 1.5	110	4.5	36
21	Swimming pool water, DF 10	124	97	n. d.	150
22	Swimming pool water, DF 20	275	260	n. d.	140
23	Swimming pool water, DF 20	289	260	n. d.	140
24	Swimming pool water, DF 20	202	150	n. d.	110
25	Swimming pool water, DF 5	56	84	n. d.	80
26	Swimming pool water, DF 5	52	74	n. d.	88
27	Swimming pool water, DF 5	54	74	n. d.	89
28	Swimming pool water, DF 5	54	n. d.	n. d.	n. d.
29	Swimming pool water, DF 5	54	67	n. d.	98
30	Swimming pool water, DF 5	55	74	n. d.	88

*LOD* limit of determination; *n. d.* not determined; *DF* dilution factor applied

Perchlorate was detected in four raw water samples, two drinking water samples, six surface water samples, and in all of the swimming pool samples. Dilution of the surface water sample (Table 4, #13) and all of the swimming pool samples removed any chromatographic interference. Samples 1, 5, 7, 8, 10, 11, 14, and 18 did not show any perchlorate signal ≥ LOD. Samples 12, 15, and 20 showed signals below LOQ but above LOD.

The indication of quantitative results from standard calibration experiments below the reporting limit of 1.5 µg/L (samples 3, 6, 16, and 17) needs to be specified including the expanded measurement uncertainty. Sample 17 showed a measurement uncertainty up to 100 % and samples 12, 15, and 20 uncertainties above 100 %. The latter ones need to be reported < 1.5 µg/L [38].

## Conclusion

An isocratic ion chromatographic method with suppressed conductivity detection was tested for routine perchlorate determinations on a low µg/L level. Validation data regarding robustness, stability of reagents, limit of quantification (LOQ), and measurement uncertainty were determined. The chromatographic set-up was applied successfully for selected drinking water, ground water, surface water, and swimming pool water samples. The equivalency of the data obtained after the applied sample preparation compared to data obtained without sample treatment was proven. After sample preparation, chromatographic resolution (R) of perchlorate and the next eluting peak always were larger than 1.3.

The perchlorate method presented in this study provides a robust, reliable, and sensitive analytical approach for perchlorate determinations at concentrations of 1.5 µg/L and above. The method could serve as an alternative, if more sophisticated IC techniques such as gradient elution, column cut, or coupling techniques (IC-MS) are not available.
